# Supplementary figures and images for: Improving gut virome comparisons using predicted phage host information
Source: mSystems. 2025 Apr 8;10(5):e01364-24. doi: 10.1128/msystems.01364-24 (PMC12090736; doi:10.1128/msystems.01364-24)

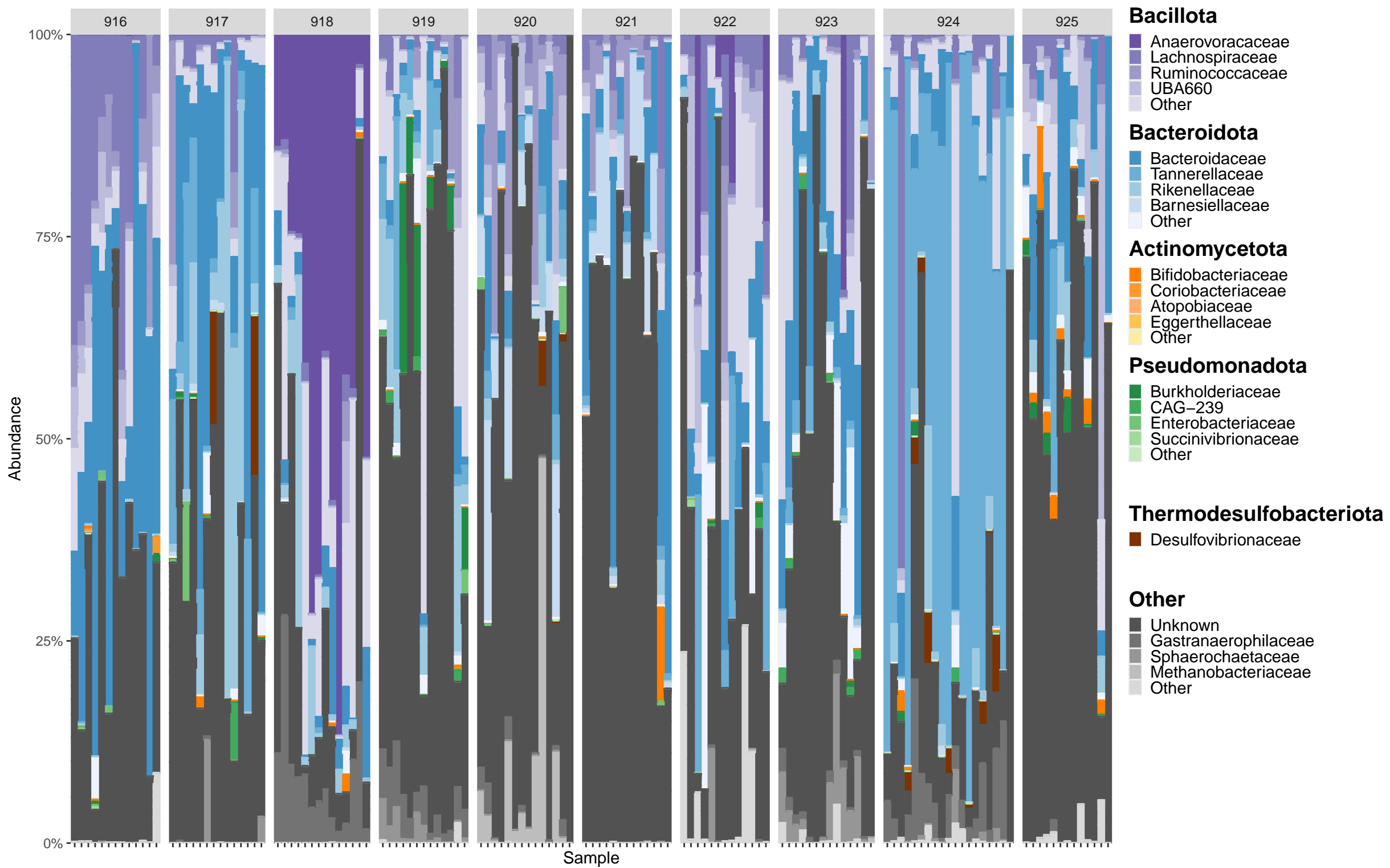

## vOTUs per PHF

vOTU Count   ● >100   ● 51-100   ● 11-50   ● 2-10   ● 1

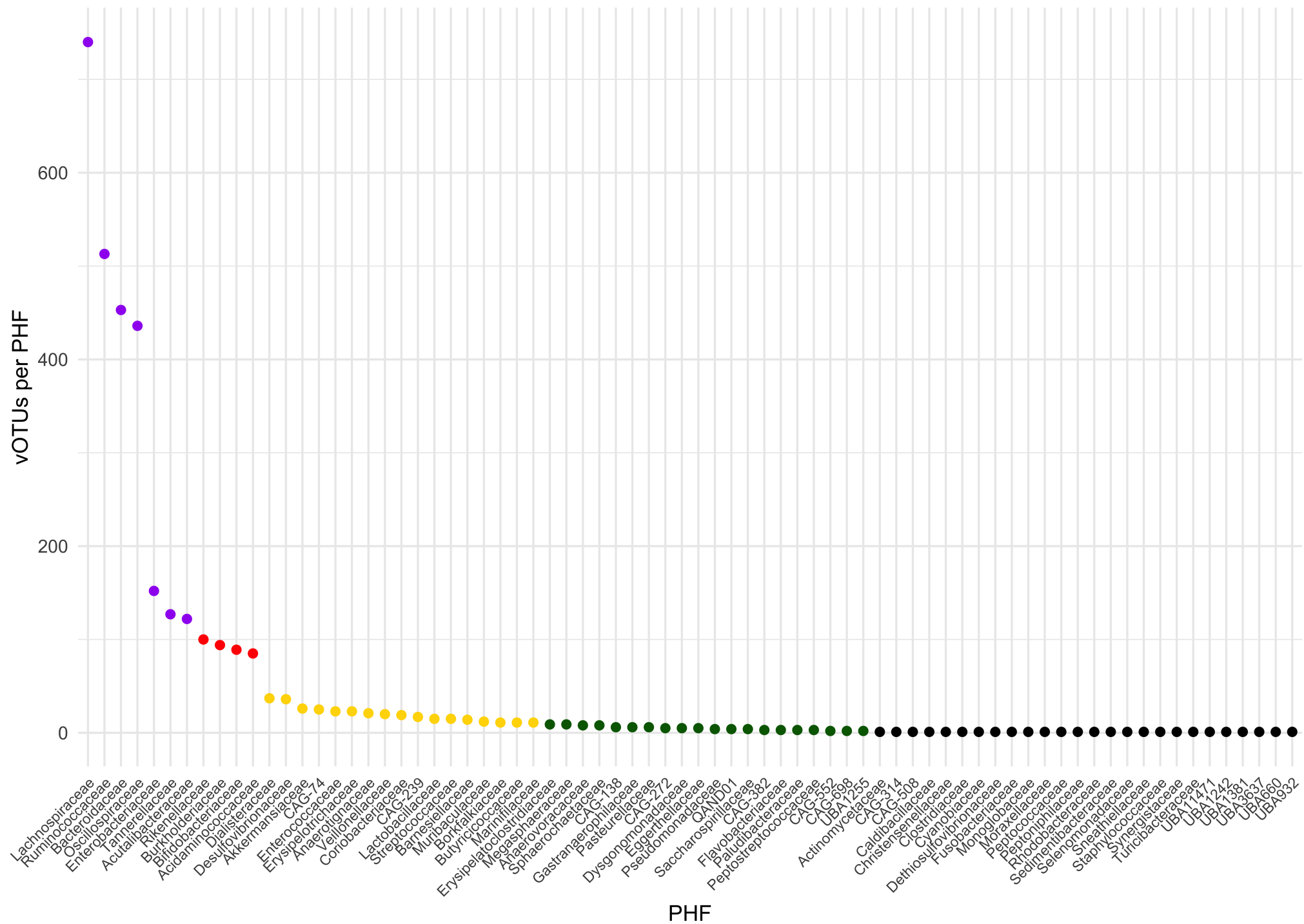

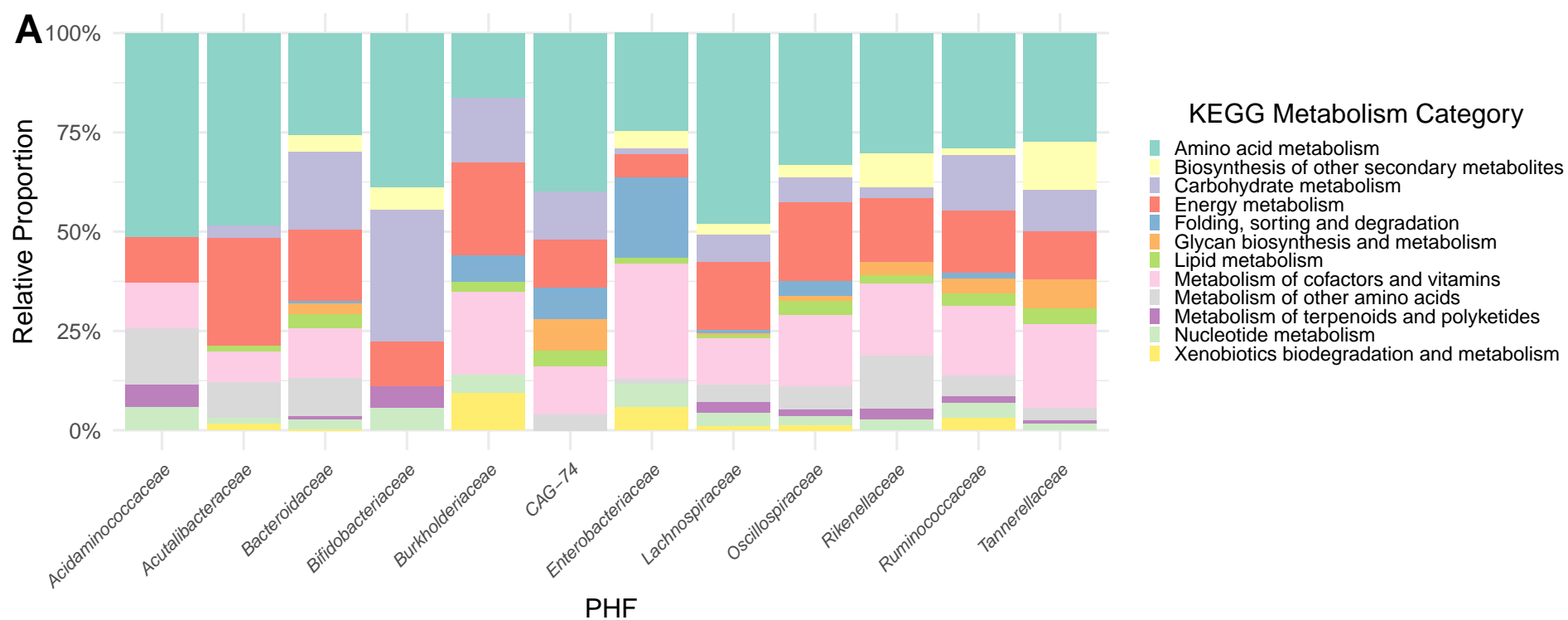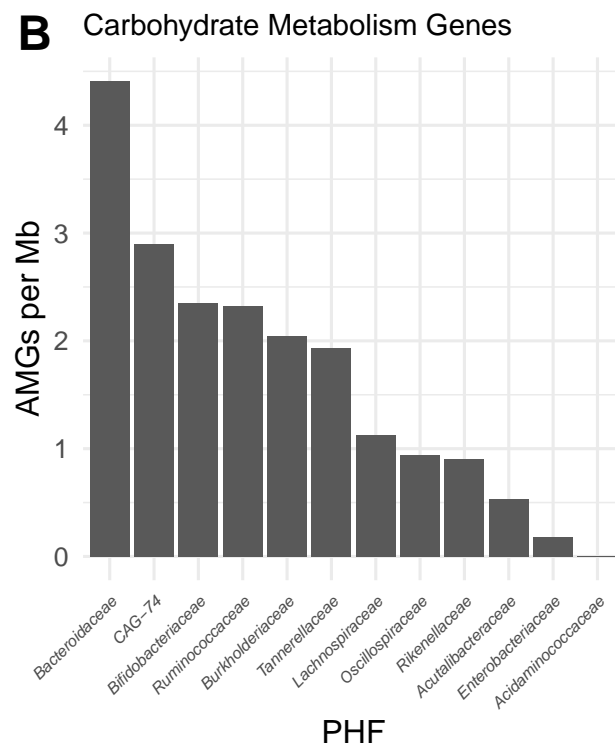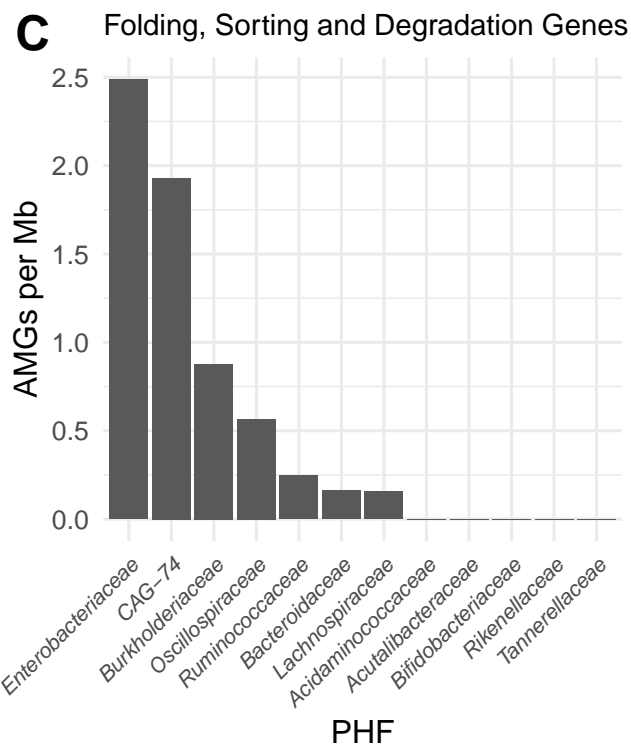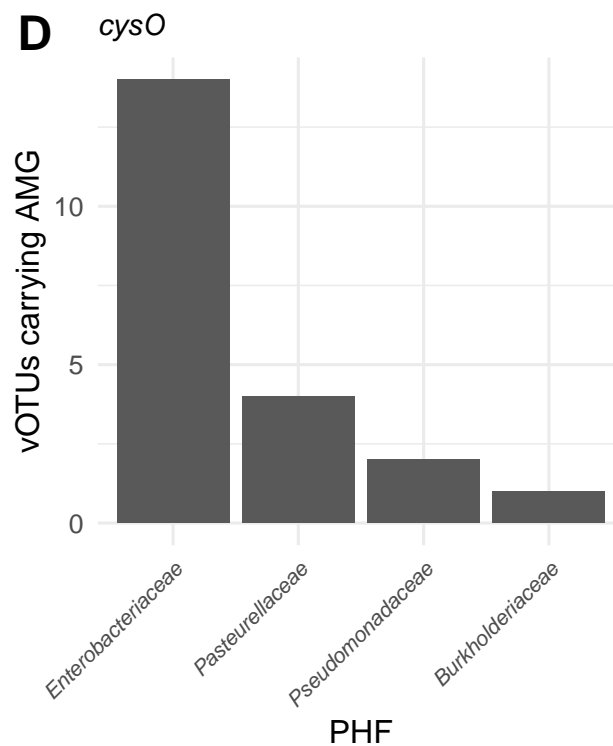

vOTU

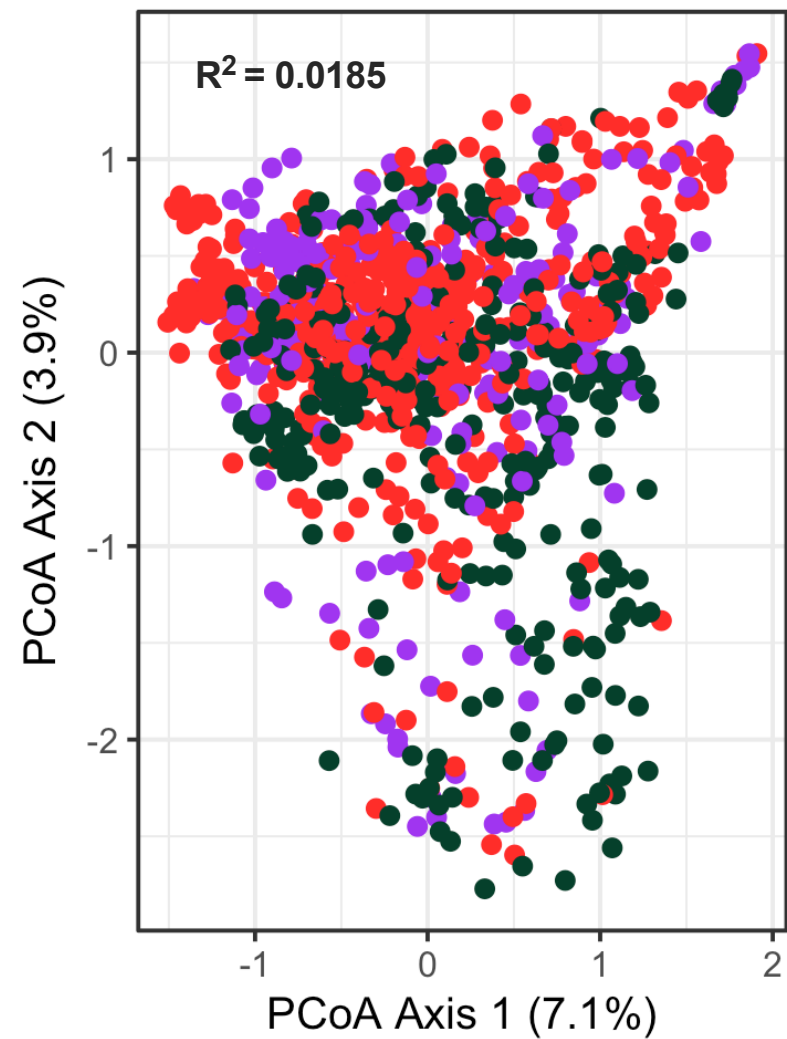

PHF

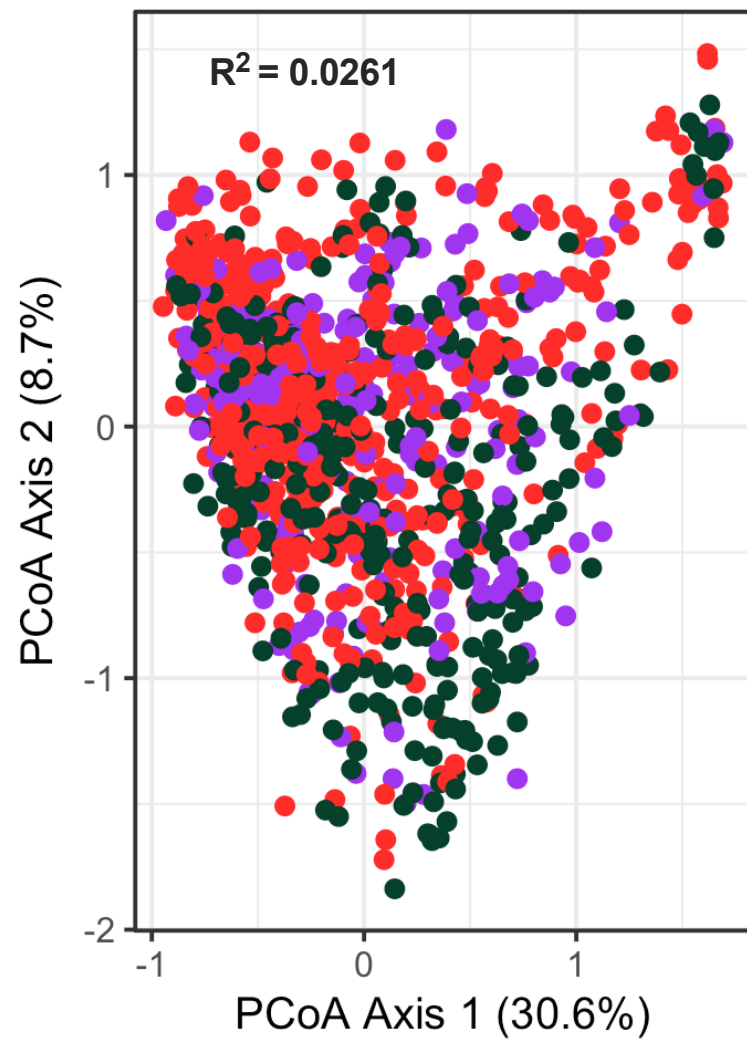

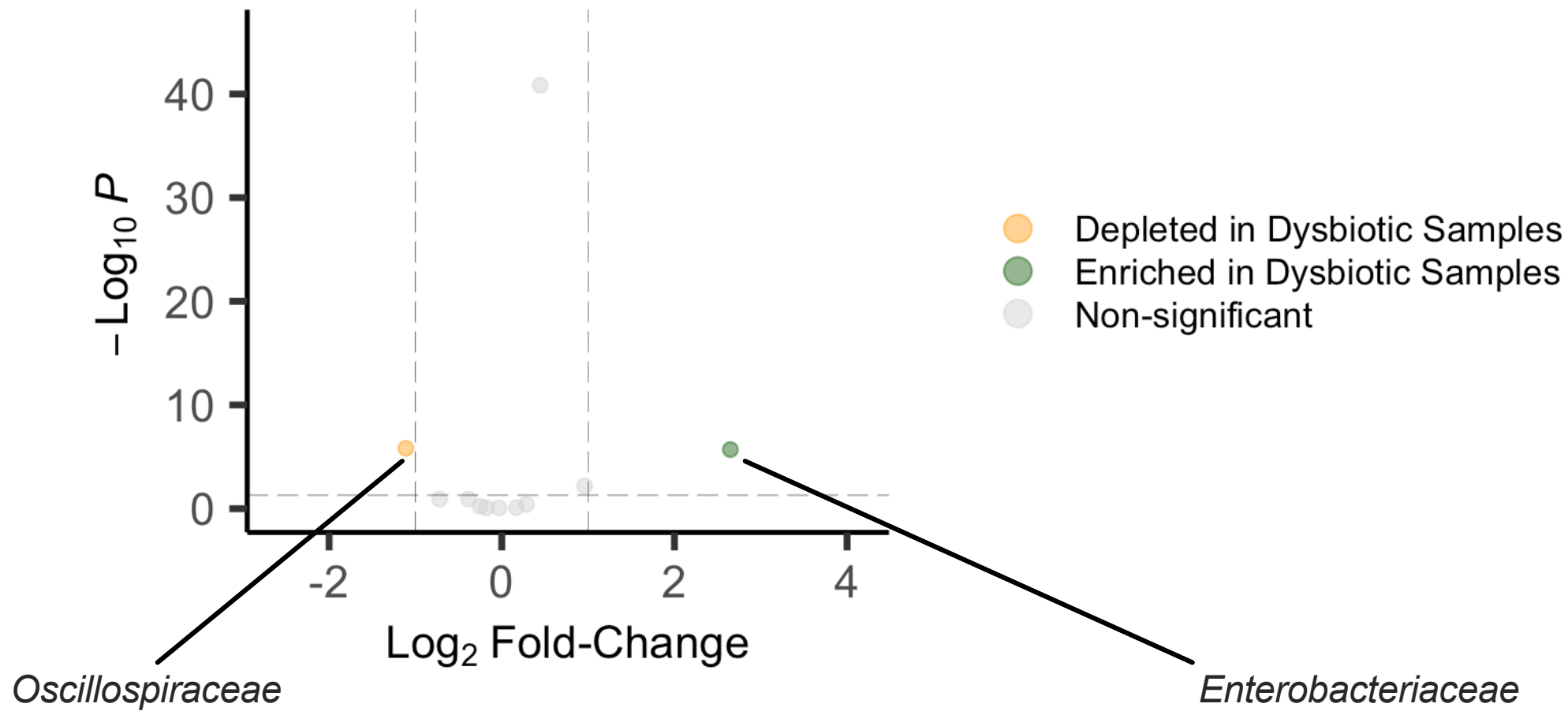

Supplement: Supplemental Figures — Figures S1 to S5. [file msystems.01364-24-s0001.pdf]
