# Supplementary material for: Improving gut virome comparisons using predicted phage host information
Source: mSystems. 2025 Apr 8;10(5):e01364-24. doi: 10.1128/msystems.01364-24 (PMC12090736; doi:10.1128/msystems.01364-24)
Supplement: Legends — for the supplemental figures and tables. [file msystems.01364-24-s0002.docx]

**Supplementary Figure 1. Longitudinal gut virome composition of 10 healthy individuals after CheckV filtering.** Data were analyzed from a previously published study of 10 healthy individuals (1). Taxonomic bar plots of virome composition at the PHF level for each individual over time, after using CheckV to keep only viral contigs >50% complete. Facet labels above the bar plots correspond to the subject IDs from the original study.

**Supplementary Figure 2. vOTU membership of PHFs.** Data were analyzed from the previously published HMP2 dataset (2). Samples with low viral read counts (< 1,500) were removed from analyses. The distribution of PHFs is based on the number of distinct vOTUs that comprises them.

**Supplementary Figure 3. AMG distribution across PHFs.** Data were analyzed from the previously published HMP2 dataset (2). Only the 12 PHFs that contained > 10 AMGs are shown here. AMGs were detected using VIBRANT, which uses KEGG annotations to assign metabolic categories. **(A)** Distribution of the AMGs found within each PHF. We then determined the number of AMGs per Mb of assembled vOTUs for each PHF, broken down by **(B)** carbohydrate metabolism genes, and **(C)** folding, sorting and degradation genes. **(D)** Number of vOTUs containing *cysO* across PHFs.

**Supplementary Figure 4. Ordination of samples based on patient diagnosis.** Data were analyzed from the previously published HMP2 dataset (2). Samples with low read counts (< 1,500) were removed from analyses. PCoA plots were generated from Bray-Curtis distances matrices using vOTUs (left) and PHFs (right). Samples are color-coded according to the diagnosis status identified in (2).

**Supplementary Figure 5. Differentially abundant bacterial families.** Data were analyzed from the previously published HMP2 dataset (2). Differentially abundant bacterial host families based on dysbiosis status. Only individuals which had both a dysbiotic and non-dysbiotic sample were included. Bacterial families which had a corresponding prevalent PHF were included for analyses. Families with an adjusted *p* value ≤ 0.05 and with a log_2_ fold-change ≥ 1 or with a log_2_ fold-change ≤ -1 were considered differentially abundant.

**Supplementary Table 1. Differentially abundant PHFs and bacterial families.** Data were analyzed from the previously published HMP2 dataset (2). Differentially abundant PHFs and bacterial host families based on dysbiosis status. Only individuals which had both a dysbiotic and non-dysbiotic sample were included. Only PHFs that were more than 50% prevalent across individuals were considered for these analyses. Bacterial families which had a corresponding prevalent PHF were included for analyses. PHFs or bacterial families with an adjusted *p* value ≤ 0.05 and with a log_2_ fold-change ≥ 1 or with a log_2_ fold-change ≤ -1 were considered differentially abundant.

**Supplementary Table 2. PHF and host family abundance correlations.** Data were analyzed from the previously published HMP2 dataset (2). Samples with low viral read counts (< 1,500) were removed from analyses. Spearman correlation coefficients were calculated based on relative abundances of PHFs and host. Significant associations were determined by *p* ≤ 0.05 after adjusting using the Benjamini-Hochberg method. All cases in which there was matching PHF and host family were included. Prevalence was calculated as the proportion of individuals which contained a PHF or host family.

**References**

1. Shkoporov AN, Clooney AG, Sutton TDS, Ryan FJ, Daly KM, Nolan JA, McDonnell SA, Khokhlova EV, Draper LA, Forde A, Guerin E, Velayudhan V, Ross RP, Hill C. 2019. The human gut virome is highly diverse, stable, and individual specific. Cell Host and Microbe 26:527–541.

2. Lloyd-Price J, Arze C, Ananthakrishnan AN, Schirmer M, Avila-Pacheco J, Poon TW, Andrews E, Ajami NJ, Bonham KS, Brislawn CJ, Casero D, Courtney H, Gonzalez A, Graeber TG, Hall AB, Lake K, Landers CJ, Mallick H, Plichta DR, Prasad M, Rahnavard G, Sauk J, Shungin D, Vázquez-Baeza Y, White RA, Bishai J, Bullock K, Deik A, Dennis C, Kaplan JL, Khalili H, McIver LJ, Moran CJ, Nguyen L, Pierce KA, Schwager R, Sirota-Madi A, Stevens BW, Tan W, ten Hoeve JJ, Weingart G, Wilson RG, Yajnik V, Braun J, Denson LA, Jansson JK, Knight R, Kugathasan S, McGovern DPB, Petrosino JF, Stappenbeck TS, Winter HS, Clish CB, Franzosa EA, Vlamakis H, Xavier RJ, Huttenhower C. 2019. Multi-omics of the gut microbial ecosystem in inflammatory bowel diseases. Nature 569:655–662.
